# Assessment of cross-cultural adaptations and patient-reported outcome measures relevant to shoulder disorders in Turkish: A systematic review using the COSMIN methodology

**DOI:** 10.1371/journal.pone.0323611

**Published:** 2025-05-27

**Authors:** Cagdas Isiklar, Gamze Cagla Sirma, Elif Turgut

**Affiliations:** 1 Department of Sports Physiotherapy, Faculty of Physical Therapy and Rehabilitation, Hacettepe University, Ankara, Türkiye; 2 Department of Physiotherapy and Rehabilitation, Faculty of Health Sciences, Fenerbahce University, Istanbul, Türkiye; 3 Department of Occupational Therapy, Faculty of Health Sciences, Fenerbahce University, Istanbul, Türkiye; Rutgers University - Newark, UNITED STATES OF AMERICA

## Abstract

**Background:**

There are many shoulder assessment outcome measures in the literature that have been studied for validity and reliability. However, there is no study examining the Turkish-adapted patient outcome measures (PROMs) on the shoulder according to the COnsensus-based Standards for the selection of health Measurement Instruments (COSMIN) checklist. In addition, there is a small number of studies that carry out this examination on the shoulder internationally. Determining the most appropriate questionnaires for clinical use will also be an important guide in patient evaluation by filling the gap in both literature and clinical aspects.

**Objective:**

Our aim in this study is to identify the valid and reliable Turkish scales used to evaluate shoulder pain and disability, to reveal how compatible these scales are with the aspects of study quality and psychometric quality according to COSMIN criteria list.

**Methods:**

A systematic search was performed in the following electronic databases: MED-LINE, Web of Science (WOS), EMBASE, SCOPUS, and ULAKBIM were searched from inception to September 2024.

**Results:**

A total of 2890 articles were found in the databases. Four articles were included additionally without a search. Then, after eliminating the duplicates, 2231 articles were included based on the title and abstract search. 63 articles were included in full-text review. After the full-text review, according to our criteria, 29 articles were included in the systematic review. According to the assessment by the COSMIN checklist, 28 articles received the final classification of “inadequate”. Only 1 article (UCLA-Tr) received the final classification of “adequate”.

**Conclusion:**

There is a wide range of Turkish PROMs available for the shoulder, with only one fully meeting COSMIN methodology criteria. However, this does not imply that other PROMs lack clinical utility. Future studies should focus on refining PROMs by incorporating MCID and PASS scores and prioritizing their responsiveness to enhance their clinical relevance. Additionally, variations in PROM performance across different patient populations highlight the need for context-specific evaluations to ensure their applicability in diverse clinical settings.

**Trial registration:**

PROSPERO registration number: CRD42022298425.

## Introduction

The prevalence of shoulder pain in the general population has been reported to range between 0.7% and 55.2%, with a median value of 16.0% [1]. Rotator cuff-related shoulder pain, tendinopathies, instabilities, and frozen shoulder are the common shoulder disorders and result in a wide range of functional disability, performance impairments, and loss of quality of life [2–5]. Recently, the advances in the management of shoulder disorders have been made through more comprehensive and accurate assessment tools in rehabilitation [6].

In addition to clinical evaluation and imaging methods, measuring treatment outcomes is an important aspect of the evaluation of patient perspective about their health status. For this purpose, patient-reported outcomes measures (PROMs) are commonly used to provide an objective measure of patients’ views of their subjective symptoms, such as quality of life, functional status, or satisfaction with treatment and disability.

More than 40 instruments are available for both generic and disease-specific use in shoulder disorders [7–10]. When choosing the best PROM developed for a specific purpose, such as shoulder disability or pain, for use in clinical practice or research, it is necessary to evaluate and present the psychometric properties of existing questionnaires such as validity, reliability, and responsiveness. Therefore, the purpose and importance of measurement features of outcome measurement tools according to the COSMIN taxonomy reflect the relevance and evaluation for any measurement tool used in any application. The predictive validity of a psychometric questionnaire is therefore the essential factor in predicting a person’s attitude [11].

The Consensus-Based Standards for the Selection of Health Status Measurement Instruments (COSMIN) group have developed a consensus-based standard for assessing the quality of studies on measurement properties and provided a methodology for conducting systematic reviews of PROMs [12–14]. To determine the methodological quality based on COSMIN, the characteristics of the PROMs are evaluated against the COSMIN checklist steps.

Previous reviews of the psychometric properties of shoulder PROMs suggested that the Western Ontario Rotator Cuff (WORC) Index, the Disabilities of the Arm, Shoulder and Hand Questionnaire (DASH), the Shoulder Pain and Disability Index (SPADI), and the Simple Shoulder Test (SST) had good evidence according to GRADE approach [15,16]. According to the GRADE approach, it is essential for these surveys to provide robust evidence. Evaluating the GRADE and COSMIN approaches in conjunction offers a comprehensive understanding of the highest level of evidence. While the GRADE approach assesses the quality of evidence for clinical decisions and recommendations, focusing on aspects such as study design, risk of bias, consistency, precision, and directness [17], the COSMIN approach emphasizes the evaluation of measurement properties of health instruments, including reliability, validity, responsiveness, and methodological rigor [18]. Together, these frameworks complement each other, providing a more holistic evaluation of evidence. About measuring internal consistency this is the extent to which items within a scale measure the same underlying construct. It indicates the homogeneity of the items. Typically assessed using Cronbach’s alpha. A value between 0.7 and 0.9 is generally considered acceptable, though this depends on the context [19]. For reliability it is extent to which an instrument consistently measures a construct across time, raters, or conditions. Test-Retest Reliability: Consistency of results when the same instrument is applied at different times. Inter-Rater Reliability: Agreement between different observers using the same tool. Intra-Rater Reliability: Consistency of the same observer measuring repeatedly. Metrics like intraclass correlation coefficient (ICC) or kappa statistics can be used for measure [20]. Lastly, validity extent to which an instrument measures what it is intended to measure. Content Validity: Whether the instrument covers all relevant aspects of the construct. Construct Validity: The degree to which the instrument aligns with theoretical expectations (e.g., correlations with related constructs). Criterion Validity: The extent to which the instrument correlates with a gold standard or an external criterion. Correlations, factor analysis, or comparison with established measures can be used for assess [21]. Almost all of the currently available instruments have been developed in English-speaking countries. To be able to administer a questionnaire in a culture or language other than the culture it was developed for or the original language, an appropriate cross-cultural adaptation process must be performed so that it retains the same meaning as the original [22]. Thus, cross-culturally adapted instruments can be used for assessment and reporting only after possible misinterpretations that may occur due to cultural or lifestyle differences are eliminated.

There are several PROMs related to shoulder diseases that have been translated into Turkish [23–51]. For Turkish speakers, the Western Ontario Shoulder Instability Index – Turkish version (WOS-Tr) is recommended for glenohumeral instability, the Rotator Cuff‑Quality of Life Scale – Turkish version (RCQOL-Tr) for rotator cuff disease, arm, shoulder, and hand disabilities [52] and the Shoulder Pain and Disability Index – Turkish version (SPADI-Tr) for non-specific shoulder pain [16]. However, there is a need for systematic reviews reporting a standardized evaluation of the psychometric properties of Turkish PROMs for shoulder diseases. Therefore, the present study aimed to systematically review the study quality and psychometric quality of Turkish PROMs for shoulder diseases using the COSMIN methodology to provide data for Turkish-speaking researchers and clinicians about which PROM is best for a specific purpose.

## Methods

### Study selection

Systematic searches were performed in the following electronic databases to identify available Turkish language tools for assessing shoulder pain and disability and to demonstrate their compatibility with the COSMIN criteria list: MED-LINE, Web of Science, EMBASE, SCOPUS, and ULAKBIM. The literature search was tailored to each database and based on the protocol suggested by the COSMIN group. The search terms and the Boolean operators used in the databases included “shoulder joint”, “shoulder related disorders”, “instruments”, and “Turkish versions”. Search was performed both in English and Turkish. There was no limitation in terms publication date or language limitation. The last search was performed in September 2024. All articles were scanned on Google Scholar using the search terms mentioned above. Details of the search strategy and related criteria are provided in S6 and S7 Tables.

Inclusion criteria of our study: Studies examining the psychometric properties of instruments originally developed in another language and translated and adapted into Turkish for the assessment of shoulder joint complex or upper extremity dysfunctions (Guidelines and rating criteria for the process of cross-cultural adaptation of self-report measures are detailed in S8 Table), all PROMs including self-report tools or interview-based questionnaires that use objective measures, only full-text articles. Exclusion criteria of our study: Instruments developed for specific groups whose primary complaint did not concern shoulder-related musculoskeletal disorders (e.g., wheelchair users and patients with cancer), And also study design is based on prognostic studies, systematic reviews, meta-analyses, abstracts from conferences, books, and theses/dissertations.

### Assessment of the methodological quality of eligible studies and data extraction

Included articles have been evaluated in terms of translation and adaptation across cultures. Results are presented according to the standards and translation guidelines developed by Guillemin et al. [53] and/or Beaton et al. [54], which involves 5 steps for cross-cultural translation and adaptation as follows: (1) first translation; (2) synthesis; (3) back translation; (4) review by a committee of experts in the field; and, (5) pretesting the final version.

Data regarding the translation and cross-cultural adaptation were extracted in order to assess the design of these studies. In addition, data on the measurement properties of the COSMIN checklist were extracted for each study [55]. After that, the cross-cultural adaptation and translation methods of each study were classified according to the COSMIN methodology. Two reviewers (C.I. & G.C.S.) independently performed the assessment of the methodological quality for each criterion of the COSMIN checklist. The COSMIN checklist was used to evaluate general methodological quality. Each criterion was rated independently by the two reviewers to ensure an unbiased assessment process. Discrepancies between reviewers were addressed through a structured discussion process. In cases where consensus could not be reached, a third reviewer (E.T.) resolved the conflicts. The final ratings were derived through a consensus process, considering input from all three reviewers. To facilitate the review process and systematically manage conflicts, the Rayyan platform (https://www.rayyan.ai/) was utilized. This platform allowed for blinded independent assessments by reviewers and provided a structured environment for resolving disagreements efficiently.

The checklist contains nine boxes with standards for good methodological quality of studies on nine different measurement properties. The quality of each step is marked positive (+) when the process is performed in accordance with quality criteria; it is marked doubtful (?) when the definition of the method is unclear and there are insufficient quantity of translators and/or back translators; or zero (0) when there is not enough information to evaluate each step [13,56].

The Grading of Recommendations, Assessment, Development, and Evaluation (GRADE) approach (S8 Table) is a systematic and transparent approach to grading the precision of evidence in systematic reviews and clinical practice guidelines and to develop and determine the strength of clinical practice recommendations. The quality rating of each measurement feature can be added directly to the relevant table. If the results of some studies are ignored when condensing the evidence and determining the overall rating of the pooled or summarized outcome for a measurement feature, those studies should also be ignored in determining the quality of the evidence [57]. In this study, we identified “high-quality studies” using the GRADE (Grading of Recommendations Assessment, Development, and Evaluation) approach. According to GRADE, specifically, these studies were identified based on their ability to deliver high-quality evidence regarding validity, consistency, and reliability, as assessed through the GRADE (Grading of Recommendations Assessment, Development, and Evaluation) approach.

In summary, we have used three sets of evaluation criteria—COSMIN, GRADE, and measurement translation suggestions—to assess the measures comprehensively: The COSMIN approach was used to evaluate the methodological quality of studies related to the development and validation of the instruments. The GRADE approach was applied to assess the overall quality of evidence for each instrument, focusing on validity, reliability, consistency, and relevance to our research question. The measurement translation suggestions were included to address the practical aspects of adapting measures for cross-cultural use, ensuring conceptual and linguistic equivalence.

## Results

**Fig 1:**
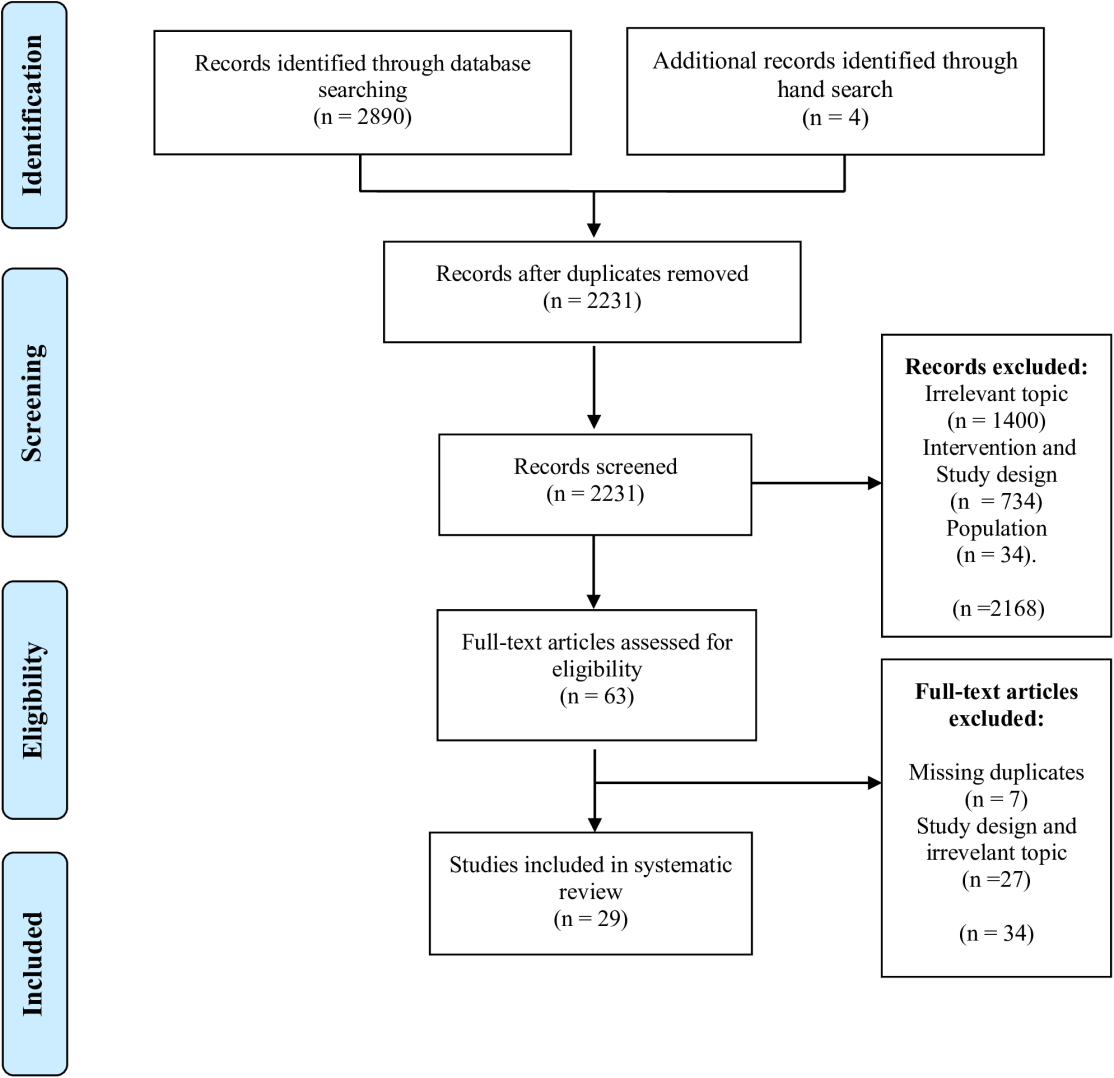
PRISMA flowchart. PRISMA, Preferred Reporting Items for Systematic Reviews and Meta-analyses.

A total of 2890 records were identified in the search. In addition, four articles were added with hand search. Based on the titles and abstracts, 63 articles were selected. Thirty-four articles that met the selection criteria were excluded after assessment due to missing duplicates and study design. Finally, 29 articles were included in this study (Fig 1). During the full-text screening, the reviewers had 93.1% agreement. The conflict on the remaining 6.9% (2 articles) was resolved after a meeting.

The questionnaires translated and adapted to Turkish were: the Western Ontario Osteoarthritis of the Shoulder Index (WOOS) [23]; the University of California Los Angeles (UCLA) Shoulder Scale [24]; the Shoulder Rating Questionnaire (SRQ) [25]; L’Insalata Shoulder Rating Questionnaire (LSRQ) [26]; the Specific Acromioclavicular Score (SACS) [27]; The Long Head of Biceps Score (LHB) [28]; the Oxford Shoulder Instability Score (OSIS) [29]; the Kerlan-Jobe Orthopaedic Clinic Shoulder and Elbow Score (KJOC-SES) [30]; the Penn Shoulder Scale (PSS) [31]; the Western Ontario Shoulder Instability Index (WOSI) [32]; the Modified Constant Murley Score (CMS) [33]; the Rotator Cuff Quality of Life (RC-QOL) [45]; the Rotator Cuff‐Quality of Life Scale (RC-QoLS) [46]; the Upper LIMB Functional Index (ULFI) [49]; the Upper Extremity Functional Index (UEFI) [35]; the American Shoulder and Elbow Surgeons Standardized Shoulder Assessment Form (ASES) [36]; the Milliken Activities of Daily Living Scale (MAS) [37]; the Quick Disability of the Arm, Shoulder, and Hand (Q-DASH) [38]; the Oxford Shoulder Score (OSS) [58]; the Shoulder Pain and Disability Index (SPADI) (adaptation, reliability and validity study) [34]; the Shoulder Pain and Disability Index (SPADI) (validation study for Turkish women) [40]; the Simple Shoulder Test (SST) [41]; the Shoulder Disability Questionnaire (SDQ) [43]; the Disability of the Arm, Shoulder, and Hand (DASH) (validity study for workers) [42]; the Disability of the Arm, Shoulder, and Hand (DASH) (adaptation, reliability and validity study) [44]; Munich Shoulder Questionnaire (validity and reliability study) [50]; the Western Ontario Rotator Cuff Index (WORC) [48]; Nottingham Clavicle Score (validity and verification study) [51]; and a comparison of the responsiveness of SDQ, SPADI, and WORC index (Responsiveness study) [47] (S1 Table).

The 5 steps of the translation process were performed in 23 studies included [23,24,26–28,30–39,43–46,48–51] (S2 Table). The results of high-quality studies are considered when determining the overall rating, the rating of the evidence is determined only by the high-quality studies (in which case we do not downgrade for the risk of bias) In the first assessment, the assessors had 88.89% agreement (32 out of 36 items). After further discussion, they reached 100% consensus. For the quality of evidence analysis, there was 100% agreement between the two reviewers (S3 Table). S4 Table presents the detailed analysis of the risk of bias and quality appraisal based on COSMIN risk of bias checklist, updated criteria for good measurement properties about questionnaires/scales.

Twenty-six questionnaires assessed internal consistency [23–26,28–43,45,46,48–51]; twenty-seven assessed criterion validity [23–45,48–51], and twenty-four analyzed reproducibility [23–28,30–33,35–46,48,49]. There is a lack of analysis among the questionnaires, mainly in relation to the properties floor/ceiling effect and responsiveness, which were verified only in nine [24,26,27,30,32,33,35,36,39] and two articles [24,50], respectively.

The SF-36 questionnaire (mental and quality of life), SPADI, and DASH was the most used comparative for validity assessment among the studies. In addition to these questionnaires, WORC, ASES, UEFI, OSS, PSS, Constant Score, and SF-12 were seen as the questionnaires used for comparison. Two of the 29 articles did not apply questionnaires/scales for comparison [46,47].

According to the assessment by the COSMIN scale, 28 articles received the final classification of “inadequate”. Only 1 article [24] received the final classification of “adequate” (**S5 Table**). Also, in the inclusion of each study and review of the studies according to the COSMIN checklist, three authors independently completed this process.

## Discussion

This systemic review aimed to evaluate the available literature regarding the psychometric properties of Turkish PROMs for shoulder diseases using the COSMIN methodology. A total of 28 distinct PROMs, out of 29 examining shoulder disorders were adapted to Turkish.

Regarding the methodological quality of the included studies according to the COSMIN checklist, there is mostly moderate or poor evidence available. The findings of this study showed that, OSS-Tr, ASES-Tr, CMS-Tr, were found proficient PROMs according to the COSMIN methodology and the UCLA-Tr was found the most appropriate PROM in this study. However, commonly used questionnaires in Turkish provide low-level evidence (S5 Table) regarding rotator cuff disease, glenohumeral instability, subacromial impingement syndrome, acromioclavicular joint instability, or other shoulder-specific upper extremity according to the COSMIN methodology.

This is the first review in the literature evaluating the available cross-cultural adaptations of shoulder PROMs using COSMIN methodology. Previously, shoulder-related PROMs translated into Portuguese were reviewed without using the COSMIN checklist [59]. It was stated that DASH and WORC are the most appropriately developed and tested questionnaires in the study conducted in Portugal [59]. In the present study, the UCLA-Tr questionnaire was determined as the most appropriate questionnaire. Most of the adapted PROMs have not been investigated for responsiveness. In addition, the clinical use of these PROMs and their suitability for patients have not been demonstrated by the minimal clinically important difference (MCID) or the patient acceptable symptom state (PASS) scores [60]. Future adapted PROMs should take these shortcomings into account.

The COSMIN checklist has been previously applied for the assessment of the original shoulder PROMs. Longo et al. investigated the methodological quality of studies in terms of the psychometric properties of PROMs for rotator cuff disease and recommended the use of WORC and RC-QOL [61]. In their review, Huang et al. deduced that the WORC had the highest ratings, followed by the DASH, SPADI, and SST [15]. Both of the two reviews have suggested that there is a need for higher quality methodological studies to evaluate the characteristics of all instruments identified in rotator cuff and shoulder pathologies. In a systematic review conducted by Villegas et al., which examined outcome measures utilized for assessing shoulder functionality, it was highlighted that a wide variety of functional assessment tools have been employed across different shoulder injuries. While this diversity enhances the range of options available for clinical practice, the authors underscored the critical need for developing high-quality outcome measures that comprehensively address diverse methodological and clinical requirements [62]. In this study, it was revealed that WORC-Tr or RC-QOL-Tr scales are available instruments for rotator cuff diseases, although they were not found to be adequate in quality according to the COSMIN methodology. It will be clinically beneficial to examine the clinical significance in both Turkish and original versions, to follow similar methods in the original patient outcome measures, and to reveal these in systematic reviews.

The main problem is that most of the studies evaluated in this review have not used any checklists for methodological quality. When designing a study evaluating the psychometric properties of a PROM, applying a checklist with the necessary standards and statistical methods will improve the methodological quality of the study [18]. In the literature, there are several methodological guidelines to evaluate the quality of PROMs, such as the Evaluating the Measurement of Patient-Reported Outcomes tool [63], the COSMIN checklist [18], and the checklist developed by Francis et al. [64]. In future studies evaluating the psychometric properties of PROMs, it is recommended to use a guide of some advantages and disadvantages suggested by Mokkink et al. [12]. Higher quality studies are needed to assess all relevant psychometric properties of current PROMs in Turkish, particularly content validity and construct validity, in order to gather stronger evidence. In addition, more research is needed to evaluate the psychometric properties of PROMs with other common musculoskeletal shoulder disorders (e.g., adhesive capsulitis).

While our initial conclusions focused on measures meeting 100% of the COSMIN criteria, we recognize that this approach may overlook the potential value of tools that, while not fully meeting all criteria, demonstrate promising properties in other aspects. For instance, the modified CMD-tr, despite lacking evidence on responsiveness, showed strong performance across most evaluated criteria. This suggests its potential utility in clinical and research settings. We also acknowledge that the absence of a detailed discussion on floor and ceiling effects does not necessarily render a measure inadequate. These effects can vary significantly depending on the population studied and may be less critical than other aspects such as validity and reliability. In this context, other PROMs used in Turkish clinical settings should not be dismissed as inadequate solely based on our findings. Instead, their classification as inadequate reflects their performance against the specific COSMIN-based criteria applied in this study. To better guide future research and practice, we propose evaluating PROMs along a continuum, highlighting their strengths, identifying areas requiring further investigation, and considering their relevance to specific populations and contexts. This approach could provide a more nuanced and practical framework for selecting and improving measurement tools.

This systematic review had three main limitations. First, theses with unpublished data on the measurement properties of the described instruments could also be included. Second, the findings of this review were not categorized by any specific population or disease and only covered the adult population. Finally, this review lacks the gray literature containing footnotes produced outside of traditional articles and distribution channels.

## Conclusion

There is a wide variety of Turkish PROMs for the shoulder, and only one specific scale was demonstrated to fit all of the parameters defined according to the COSMIN methodology. The findings of the current study regarding the psychometric properties of PROMs and the quality of the studies should be assessed in future studies using MCID and PASS scores. Furthermore, special attention should be given to improving the responsiveness of PROMs, as this is a critical aspect of their utility in clinical practice. It should also be noted that the performance of these questionnaires may vary across different patient populations, emphasizing the importance of evaluating their applicability and relevance in diverse clinical contexts. Additionally, it is important to highlight that the classification of PROMs as inadequate based on COSMIN criteria does not necessarily imply that they are clinically unusable. The definitions of inadequacy in this study are rooted in methodological parameters, which may differ from the practical utility of these tools in real-world clinical settings.

## Supporting information

S1 Table Characteristics of the included PROMs evaluated in the systematic review. (DOCX)

S2 Table Cross-cultural adaptation steps based on guideline recommendations. (DOCX)

S3 Table GRADE assessment of the quality of evidence for measurement properties. (DOCX)

S4 Table COSMIN Risk of Bias ratings for each included PROM. (DOCX)

S5 Table Summary of psychometric properties and COSMIN quality assessment. (DOCX)

S6 Table English and Turkish search strategies used in the review process. (DOCX)

S7 Table Criteria and rating system for cross-cultural adaptation of self-report measures. (DOCX)

S8 Table Overview of the GRADE framework and downgrading rules. (DOCX)

S1 Checklist PRISMA 2020 checklist (2 and 3). (DOCX)
