# Supplementary material for: Assessment of cross-cultural adaptations and patient-reported outcome measures relevant to shoulder disorders in Turkish: A systematic review using the COSMIN methodology
Source: PLoS One. 2025 May 27;20(5):e0323611. doi: 10.1371/journal.pone.0323611 (PMC12111439; doi:10.1371/journal.pone.0323611)
Supplement: S1 Table — (DOCX) [file pone.0323611.s001.docx]

**S1 Table. Characteristics of the Included PROMs**

| PROM | Author / Year | Population | N | Age  (Mean ± SD) | Mode of administration | Number of domains | | Number of items | Subscales | Response options | Recall period | Range of scores / scoring |
| --- | --- | --- | --- | --- | --- | --- | --- | --- | --- | --- | --- | --- |
| NCS-Tr | Gülcü et al., 2023 | Patients with clavicle fractures | 68 | 37.1±13.3 | Self-reported measure | 1 | 10 | | -Pain  -Strength and lifting capacity  -Cosmetic Satisfaction  -Movement of the shoulder  -Tingling and numbness | Likert scale ranging from 0 to 5 | 7-10 days | 20 to 100 |
| MSQ-Tr | Kocamaz et al., 2022 | Patients with shoulder problems | 180 | 44.3±11.6 | Self-reported measure | 3 | 30 | | -Introduction  -Objective  -Subjective | Numeric scale 0 to 10 |  | 0 to 50 |
| WOOS – Tr | Ozal et al., 2021 | Patients with shoulder osteoarthritis | 67 | 61.5 ± 8.7 | Self-reported measure | 4 | 19 | | -Physical symptoms  -Sports/ recreation/ work  -Life style  -Emotional well being | 10-cm visual analog scale | 2 days after | 0 to 1.900 |
|  |  |  |  |  |  |  |  | |  |  |  |  |
| UCLA – Tr | Buyukdogan et al., 2021 | Patients with shoulder disorders | 91 | 46.0 ± 13.7 | Self-reported measure | 5 | 5 / 26 | | -Pain  -Function  -Active forward flex  -Strength of forward flex  -Overall satisfaction | One item for every question | 3-7 days (mean 5.2 days) after | 0 to 35 |
| SRQ – Tr | Ciftci et al., 2021 | Patients with various shoulder pain | 122  Working Group (N=72)  Non-working Group (N=50) | Working Group:  55.36 ± 14.20  Non-working Group:  54.80 ± 10.07 | [Self-reported measure](https://methods.sagepub.com/reference/encyclopedia-of-survey-research-methods/n523.xml#:~:text=Self%2Dreported%20measures%20are%20measures,semantic%20differentials%20are%20self%2Dreport.) | 5 | 19 | | -Global assessment  -Pain  -ADL  -Sports/ recretional activites  -Work | 10 cm - visual analogue scale +  Five-choice questions (scored from 1 to 5) | least 3-days after | 17 to 100 |
| LSRQ – Tr | Yasar et al., 2021 | Patients with shoulder pain | 90 | 44.37 ± 14.94 | Self-reported measure + interwieved based | 7 | 21 | | -Global  -Pain  -Daily activites  -Sports/recreation  -Work | 10 cm visual analog scale + multiple choice questions with a scoring from 1 to 5 + goniometer | 3-7 days after | 17 to 100 |
| SACS – Tr | Oguzkaya et al., 2021 | Patients who were diagnosed with acute or  chronic ACJ instability or symptomatic ACJ arthritis were included in  the study. | 78 | 38.2 ± 12.6 | Self-reported measure | 3 | 21 | | -Pain,  -Function  -Quality of life | 10-cm visual analog scale  Numeric scale 0 to 10 | 2 weeks after | 0 to 150 |
| LHB Score – Tr | Najafov et al., 2021 | Patients with biceps pathologies | 62 | 62.53 ± 10.15 | Self reported measure + interviewed based | 3 | 7 | | -Pain/cramps  -Cosmesis  -Elbow-flexion strength | Numeric scale | 2 days after | 0 to 100 |
| OSIS – Tr | Sonmezer et al., 2020 | Patients with shoulder instability | 118 | 52 ± 14 | Self-reported measure | 1 | 12 | | assess the functional status and quality of life of patients with shoulder instability | Five-choice questions | 2 days after | 0-48 |
| WOSI - Tr | Basar et al., 2017 | Patients with shoulder instability | 74 | 33.9 ± 13.3 | Self-reported measure | 4 | 21 | | -Physical symptoms  -Sports/ recreation/ work  -Life style  -Emotional well being | 0–10 numeric scoring system | 3 days after | 0 to 2.100 |
|  |  |  |  |  |  |  |  | |  |  |  |  |
| KJOC-SES – Tr | Turgut et al., 2018 | Overhead athletes | 123 | 23.1 ± 5.06 | Self-reported measure | 1 | 10 | | measure functional status of the upper extremities in overhead athletes | 100-mm visual analog scale | 7 days after | The total score is calculated as an average of the total scores of the 10 questions. |
| PSS – Tr | Kanik et al., 2018 | Patients with shoulder dysfunctions | 97 | 52.8 ± 13.4 | Self-reported measure | 3 | 24 | | -Pain  -Satistfaction  -Function | 10-point visual analog scale +  4-point Likert scale | 3 days after | 0 to 100 |
| The modified CMS – Tr | Celik et al., 2016 | Patients with various shoulder pathologies | 30 | 59.5 ± 13.5 | Self reported measure + interviewed based | 4 | - | | -Pain  -ADL  -Strenght  -ROM | 0–10 numeric scoring system | 2 hours after | 0 to 100 |
| RC-QOL – Tr | Cinar-Medeni et al., 2015 | Rotator cuff-impaired (11 impingement, 6 arthroscopic rotator cuff repair, and 13 partial thickness rotator cuff tear) patients | 30 | 47.5 ± 13.9 | Self-reported measure | 5 | 34 | | -Symptoms and physical complaints  -Work or profession  -Recreation and sports  -Life style  Social and emotional aspects of shoulder problems | 100-mm visual analog scale | 2 days after | 0 to 3.400 |
| RC-QoLS – Tr | Gunes et al., 2015 | RC tear on one shoulder scheduled for surgery. | 54 | 56 ± NR | Self-reported measure | 5 | 34 | | -Symptoms and physical complaints  -Work or profession  -Recreation and sports  -Life style  Social and emotional aspects of shoulder problems | 100-mm visual analog scale | 7 days after | 0 to 3.400 |
| WORC – Tr | El et al., 2006 | Patients with rotator cuff disase | 72 | 54.9 ± 9.9 | Self-reported measure | 5 | 21 | | -Total score  -Physical symptoms  -Sports/recreation  -Work  -Life style  -Emotions | 100-mm visual analog scale | 2–7 days (mean 2.9 days) after | 0 to 2.100 |
| ULFI – Tr | Tonga et al., 2015 | Subacute patients with upper extremity muscoskeletal disorders | 127 | 49.1 ± 16.6 | Self-reported measure | 1 | 25 | | assess activity limitations and participation restrictions | “Yes (1)”, “Half (0,5)”, “No (0)” | 3 days after | 0 to 100 |
| UEFI – Tr | Aytar et al., 2015 | Patients who were diagnosed with SAIS enrolled to this study | 93 | 56.00 ± 13.81 | Self-reported measure | 1 | 20 | | -Shoulder function | Likert scale ranging from 0 to 4 | 5 days after | 0 to 80 |
| ASES – Tr | Celik et al., 2013 | Patients suffering from shoulder complaints | 75 | 48.2 ± 13.4 | Self-reported measure + Interviewed based | 2 | 11 | | -Pain  -Function | 4-point Likert scale + visual analogue scale of 0 to 10 | 3 to 7 days after | 0 to 100 |
| MAS – Tr | Akel et al., 2012 | Patients with different upper extremity problems referred to the outpatient | 99 | 41.21 ± 12.08 | Self-reported measure | 6 | 47 | | Meal preparation and eating Personal hygiene  Dressing  Object manipulation Housecleaning and laundry Other activities | Likert scale ranging from 1 to 5 + 1 to 3 | 7 days after | 47 to 235 |
| Q - DASH - Tr | Dogan et al., 2011 | Patients with idiopathic Carpal Tunnel Syndrome and  patients with musculoskeletal disorders of the upper limb | 69 | 52.43 ± 11.63 | Self-reported measure | 3 | 11 | | -Disability/ symptom  -Work  -Sport/ performin arts | 100-mm visual analog scale? +  5-point Likert scale (1 to 5) | 2 - 3 days after | 0 to 55 |
| DASH – Tr for industry workers | Kitis et al., 2009 | Industry workers with upper-extremity musculoskeletal complaints. | 240 | 27, 32 ± 7,59 | Self-reported measure | 2 | 30 | | -Work component  -Functional component | 5-point Likert scale (1 to 5) | 15 days after | 0 to 100 |
| DASH - Tr | Duger et al., 2006 | Patients with upper extremity complaints | 134 | 49.2 ± 14 | Self-reported measure | 3 (+ 2 are optional) | 30 | | -Disability and symptoms | 5-point Likert scale | 7 days after | 0 to 100 |
| OSS – Tr | Tugay et al., 2011 | Patients with shoulder problems | 84 | 49.26 ± 11.92 | Self-reported measure | 2 | 12 | | -Pain  -Quality of life. | scored between 0 and 4. | 2 days after | 0 to 48 |
| SPADI – Tr for Turkish women | Bicer et al., 2010 | Women patients with shoulder pain | 101 | 53.10 ± 8.52 | Fill under supervision | 2 | 13 | | -Pain  -Disability: difficulty an individual has with various activities of Daily living requiring the use of upper extremities | 10-cm visual analog scale | 5-7 days after | 0 to 130 |
| SPADI – Tr | Bumin et al., 2008 | Patients with shoulder problems | 140 | 60.31 ± 13.02 | Self-reported measure | 2 | 13 | | -Pain  -Disability | visual analog scales from 0 to 10 |  | 0 to 130 |
| SDQ - Tr | Ozsahin et al., 2008 | Patients with shoulder problems | 80 | 51.2 ± 11.4 | Self-reported measure | 1 | 16 | | -Functional status limitation and pain | ‘yes’, ‘no’ or ‘not applicable | 7 days after | 0 to 100 |
| A comparison of the responsiveness of SDQ, SPADI and WORC index | Dogu et al., 2013 | Patients with shoulder pain lasting more than three months and diagnosed Shoulder Impingement Syndrome | 64 | 62.53 ± 10.15 |  |  |  | |  |  |  |  |
| SST – Tr | Ayhan et al., 2010 | Patients with shoulder problems | 65 | 49.2±14.0 | Self-reported measure | 1 | 12 | | -Symptoms and functions | Yes / No (1 / 0) | 7 days after | 0 to 12 |

Empty cells indicate that no information was available on this item
