# Supplementary material for: Assessment of cross-cultural adaptations and patient-reported outcome measures relevant to shoulder disorders in Turkish: A systematic review using the COSMIN methodology
Source: PLoS One. 2025 May 27;20(5):e0323611. doi: 10.1371/journal.pone.0323611 (PMC12111439; doi:10.1371/journal.pone.0323611)
Supplement: S2 Table — (DOCX) [file pone.0323611.s002.docx]

**S2 Table. Cross-Cultural Adaptations of the Shoulder Questionnaires Adapted Into Turkish That Used the Translation-Based Approach Related To The Guidelines For the Process of Cross-Cultural Adaptation of Self-Report Measures.**

| Studies | Translation | Synthesis | Back Translation | Expert Committe Review | Pretesting |
| --- | --- | --- | --- | --- | --- |
| NCS-Tr | + | + | + | + | + |
| MSQ-Tr | + | + | + | + | + |
| WOOS – Tr | + | + | + | + | + |
| UCLA – Tr | + | + | + | + | + |
| SRQ – Tr | + | ? | + | + | + |
| LSRQ – Tr | + | + | + | + | + |
| SACS – Tr | + | + | + | + | + |
| LHB Score - Tr | + | + | + | + | + |
| OSIS – Tr | + | ? | + | + | + |
| KJOC-SES – Tr | + | + | + | + | + |
| PSS – Tr | + | + | + | + | + |
| WOSI – Tr | + | + | + | + | + |
| The modified CMS – Tr | + | + | + | + | + |
| RC-QOL- Tr | + | + | + | + | + |
| RC-QoLS-Tr | + | + | + | + | + |
| ULFI – Tr | + | + | + | + | + |
| UEFI – Tr | + | + | + | + | + |
| ASES – Tr | + | + | + | + | + |
| A comparison of the responsiveness of SDQ, SPADI and WORC index | 0 | 0 | 0 | 0 | 0 |
| MAS – Tr | + | + | + | + | + |
| Q-DASH - Tr | + | + | + | + | + |
| OSS – Tr | + | + | + | + | + |
| SPADI – Tr Turkish women | 0 | 0 | 0 | 0 | 0 |
| SST – Tr | + | + | + | ? | + |
| DASH – Tr for industry workers | 0 | 0 | 0 | 0 | 0 |
| SPADI – Tr | + | + | + | + | + |
| SDQ- Tr | + | + | + | + | + |
| DASH - Tr | + | + | + | + | + |
| WORC – Tr | + | + | + | + | + |
