# Supplementary material for: Assessment of cross-cultural adaptations and patient-reported outcome measures relevant to shoulder disorders in Turkish: A systematic review using the COSMIN methodology
Source: PLoS One. 2025 May 27;20(5):e0323611. doi: 10.1371/journal.pone.0323611 (PMC12111439; doi:10.1371/journal.pone.0323611)
Supplement: S3 Table — (DOCX) [file pone.0323611.s003.docx]

**S3 Table. Grade Approach**

|  | UCLA - Tr | | | CMS - Tr | | | ASES - Tr | | | OSS - Tr | |
| --- | --- | --- | --- | --- | --- | --- | --- | --- | --- | --- | --- |
|  | **Overall rating** | **Quality of evidence** | **Overall rating** | | **Quality of evidence** | **Overall rating** | | **Quality of evidence** | **Overall rating** | | **Quality of evidence** |
| Structural validity | ? | Low | N | | N | N | | N | N | | N |
| Internal consistency | + | Low | + | | Low | + | | Moderate | + | | High |
| Cross-cultural validity | ? | Low | N | | N | N | | N | N | | N |
| Measurement invariance | ? | Low | N | | N | N | | N | N | | N |
| Reliability | + | Moderate | + | | Moderate | + | | Moderate | + | | High |
| Measurement error | + | Moderate | + | | Moderate | + | | Moderate | + | | High |
| Criterion validity | + | Moderate | + | | Moderate | + | | Moderate | + | | Moderate |
| Construct validity | ? | Moderate | + | | Moderate | - | | Moderate | ? | | Moderate |
| Responsiveness | + | Moderate | N | | N | N | | N | N | | N |

N: not available
