# Supplementary material for: Assessment of cross-cultural adaptations and patient-reported outcome measures relevant to shoulder disorders in Turkish: A systematic review using the COSMIN methodology
Source: PLoS One. 2025 May 27;20(5):e0323611. doi: 10.1371/journal.pone.0323611 (PMC12111439; doi:10.1371/journal.pone.0323611)
Supplement: S4 Table — (DOCX) [file pone.0323611.s004.docx]

**S4 Table. Risk of Bias and Quality Appraisal Based On Cosmın Risk of Bias Checklist, Updated Criteria For Good Measurement Properties.**

| PROM | Structural Validity | Internal Consistency | Cross-cultural validity | Reliability | Measurement Error | Criterion validity | Construct validity | Responsiveness |
| --- | --- | --- | --- | --- | --- | --- | --- | --- |
| NCS-Tr | V | V |  | V |  |  | V |  |
| MSQ-Tr | A | V | A | V |  | V | V | V |
|  |  |  |  |  |  |  |  |  |
| WOOS – Tr | V | V |  | V |  | V | V |  |
| UCLA – Tr | V | V | V | V | V | V | V | V |
| SRQ – Tr |  | V |  | V |  | A | A |  |
| LSRQ – Tr | V | V |  | A | V | V | V | I |
| SACS – Tr | V |  |  | V |  | V | V |  |
| LHB Score – Tr |  | A |  | V |  | V | V |  |
| OSIS – Tr |  | V |  | V |  | V | V |  |
| KJOC-SES – Tr | V | V |  | V | V | V | V |  |
| PSS – Tr | A | V |  | V |  | V | V |  |
| WOSI – Tr | V | V |  | V |  | V | V |  |
| The modified CMS – Tr | V | V | V | V | V | V | V |  |
| RC-QOL – Tr |  | V | V | V | V | V | V |  |
| RC-QoLS – Tr |  | V |  | V |  |  | V |  |
| ULFI – Tr | V | V |  | V | V | V | V |  |
| UEFI – Tr | V | V |  | V |  | V | V |  |
| ASES – Tr | V | V | V | V | V | V | V |  |
| A comparison of the responsiveness of SDQ, SPADI and WORC index |  |  |  |  |  |  |  | V |
| MAS – Tr |  | V |  | V |  | V | V |  |
| Q-DASH - Tr |  | V | V | V |  | V | V |  |
| OSS – Tr | V | V | V | V | V | V | A |  |
| SPADI – Tr for Turkish women | V | V |  | V |  | V | V |  |
| SST – Tr |  | V |  | V |  | V | V |  |
| DASH - Tr for industry workers | V | V |  | V |  | V | V |  |
| SPADI – Tr | V | V | A |  |  | V | V |  |
| SDQ- Tr |  | V | A | V |  | V | V |  |
| DASH- Tr |  |  |  | V |  | V | V |  |
| WORC – Tr |  | V | V | V |  | V | V |  |

Empty cells indicate that no information was available on this item

PROM patient-reported outcome measure, V very good, A adequate, D doubtful, I inadequate
