# Supplementary material for: Assessment of cross-cultural adaptations and patient-reported outcome measures relevant to shoulder disorders in Turkish: A systematic review using the COSMIN methodology
Source: PLoS One. 2025 May 27;20(5):e0323611. doi: 10.1371/journal.pone.0323611 (PMC12111439; doi:10.1371/journal.pone.0323611)
Supplement: S5 Table — (DOCX) [file pone.0323611.s005.docx]

**S5 Table. Psychometric Measures of the Questionnaires**

| PROM Tool | Modifications | Internal Consistency | Criterion Validity | Reproducibility | Floor/Ceiling effect | Responsiveness | COSMIN quality |
| --- | --- | --- | --- | --- | --- | --- | --- |
| NCS-Tr | Without changes | 0.933 | OSS: 0.86^a^  DASH: -0.89^a^  SF-36: 0.59-0.81^a^ | 0.82 (ICC) | NR | NR | Inadequate |
| MSQ-Tr | Adaptations of terms | 0.73-0.96 | DASH: 0.70^a^  SPADI: 0.65^a^ | 0.91 (ICC) | NR | 2.36 | Inadequate |
| WOOS - Tr | Without changes | 0.92 | WORC: 0.84^b^  SPADI: 0.78^b^  ASES: -0.75^b^ | 0.97 (ICC) | NR | NR | Inadequate |
| UCLA - Tr | Without changes | + | SST: 0.75^a^  ASES: 0.78^a^ | 0.86-0.94 (ICC)  0.4-1.6 (SEM)  1.8-3.5 (MDC) | ^d^ | 3.22-4.31 (ES) | Adequate |
| SRQ - Tr | Adaptations of terms | 0.81-0.77 | DASH-T: 0.78^b^ | 0.97 (ICC) | NR | NR | Inadequate |
| LSRQ - Tr | Without changes | 0.89 | UEFI: 0.64^b^  OSS: -0.76^B^  SPADI: -0.75^b^  PSS: -0.49^b^ | 0.65-0.95 (ICC) | ^d^ | NR | Inadequate |
| SACS - Tr | NR | NR | OSS: 0.64^b^  SPADI: 0.77^b^  ASES: -0.68^b^ | 0.98 (ICC) | ^d^ | NR | Inadequate |
| LHB Score - Tr | Without changes | 0.64 | ASES: 0.52^b^  mCMS: 0.51^b^ | 0.94 (ICC) | NR | NR | Inadequate |
| OSIS - Tr | Adaptations of terms | 0.87 | SF-36: -0.02, -0.59^b^ | NR | NR | NR | Inadequate |
| KJOC - SES - Tr | NR | 0.94 | DASH: -0.64^a^  ASES: 0.83^a^ | 0.93 (ICC)  0.91-3.16 (SEM) | ^d^ | NR | Inadequate |
| PSS - Tr | Adaptations of terms | 0.81 | Constant Score: 0.65^a^  ASES: 0.78^a^  WORC: -0.77^a^ | 0.78-0.90 (ICC) | NR | NR | Inadequate |
| WOSI - Tr | Without changes | 0.91 | Rowe Score: -0.57^a^  OSIQ: 0.74^a^  DASH: 0.67^a^  WORC: 0.89^a^ | 0.97 (ICC) | 0%-0%^d^ | NR | Inadequate |
| The Modified CMS - Tr | Without changes | 0.88 | ASES: 0.48^b^  SF-12: 0.35-0.05^b^ | 0.87 (ICC)  6.0 (SEM)  16.4 (MDC) | ^d^ | NR | Inadequate |
| RC-QOL - Tr | Without changes | 0.83-0.98 | SPADI: 0.90^a^  WORC: 0.85^a^ | 0.71-0.97 (ICC)  4.2%-17.92% (SEM) | NR | NR | Inadequate |
| RC-QoLS - Tr | NR | 0.89-0.98 | NR | 0.80-0.97 (ICC) | NR | NR | Inadequate |
| ULFI - Tr | Adaptations of terms | 0.87 | DASH: 0.68^a^ | 0.72 (ICC)  2.94 (SEM)  5.35 (MDC) | NR | NR | Inadequate |
| UEFI - Tr | Adaptations of terms | 0.89 | SPADI: -0.61^A^  Quick DASH: -0.63^A^  SF-36 (Mental): -0.05^A^ | 0.80 (ICC) | ^d^ | NR | Inadequate |
| ASES - Tr | Adaptations of terms | 0.88 | SPADI: -0.82^a^  SF-36 (Mental): 0.64^a^ | 0.86-0.95 (ICC)  1.63-2.51 (SEM) | ^d^ | NR | Inadequate |
| *A comparison of the responsiveness of SDQ, SPADI and WORC index | - | - | - | - | - | SDQ: 0.94 (ES)  SPADI: 1.55 (ES)  WORC: 1.37 (ES) | - |
| MAS – Tr | Adaptations of terms | 0.96 | DASH: -0.35- -0.64  Grip Strength: 0.25-0.50 | 0.76-0.84 (ICC) | NR | NR | Inadequate |
| Q-DASH - Tr | Without changes | 0.84-0.92 | VAS: -0.46- 0.44^b^  Hand Grip Strength: -0.35-0.15^b^  Pinch Grip Strength: -0.54- -0.35^b^  Boston Symptom Severity Subscale: 0.28-0.68^b^  Boston Functional Status Subscale: 0.35- 0.77^b^ | 0.77-0.93 (ICC) | NR | NR | Inadequate |
| OSS – Tr | Without changes | 0.92 | SPADI: -0.7^a^  SF-36 (Physical): 0.6^a^ | 0.93-0.99 (ICC)  0.76 (SEM) | ^d^ | NR | Inadequate |
| SPADI – Tr for Turkish Women | - | 0.94 | VAS: 0.65^b^  HAQ: 0.67^b^ | 0.92 (ICC) | NR | NR | Inadequate |
| SST – Tr | Without changes | 0.72 | DASH: 0.43-0.45^a^ | 0.75 (ICC) | NR | NR | Inadequate |
| DASH – Tr for industry workers | - | 0.91 | SF-36: -0.33- -0.82^A^ | 0.92 (ICC) | NR | NR | Inadequate |
| SPADI – Tr | Adaptations of terms | 0.83 | SF-36: -0.31- -0.59^A^  DASH: 0.35-0.59^A^ | NR | NR | NR | Inadequate |
| SDQ- Tr | Without changes | 0.76 | CMS: -0.26- -0.47 | 0.88 (Pearson Correlation) | NR | NR | Inadequate |
| DASH - Tr | Adaptations of terms | NR | SF-36: -0.53- 0.59^c^ | 0.79-0.91 (ICC) | NR | NR | Inadequate |
| WORC – Tr | Without changes | 0.92 | UCLA: -0.59^a^  Constant Score: -0.63^a^  SF-36: -0.35- -0.67^a^ | 0.96-0.98 (ICC) | NR | NR | Inadequate |

a= Cronbach’s alpha (or coefficient alpha)

ICC= Intraclass Correlation

NR= Not reported

^a^Pearson’s Correlation

^b^Spearman’s Correlation

^c^Verified through ICC

^d^No effect

*SDQ, SPADI and WORC Index have excellent ability to detect clinically significant changes over time in patient with SIS.

Considering these three indexes, the SDQ and the SPADI are more suited for a rapid assessment in a short period of time, whereas the WORC index is preferred in cases where a more deailed assessment is needed, including the psychological impact of the functional status of the patient
