# Supplementary material for: Assessment of cross-cultural adaptations and patient-reported outcome measures relevant to shoulder disorders in Turkish: A systematic review using the COSMIN methodology
Source: PLoS One. 2025 May 27;20(5):e0323611. doi: 10.1371/journal.pone.0323611 (PMC12111439; doi:10.1371/journal.pone.0323611)
Supplement: S6 Table — (DOCX) [file pone.0323611.s006.docx]

**S6 Table. English and Turkish search strategies used in the review process.**

| Search Strategy in English | Query |
| --- | --- |
| 1 | (“shoulder”[MeSH] OR “shoulder impingement syndrome”[MeSH] OR "shoulder pain"[MeSH] OR “shoulder joint”[MeSH] OR “rotator cuff”[MeSH] OR “shoulder disorder*”[Text Word] OR “shoulder instability”[Text Word] OR “joint instability”[MeSH] “subacromial”[Text Word] OR “unspecified shoulder pain”[Text Word] OR “adhesive capsulit*”[Text Word] OR “frozen shoulder”[Text Word] OR "bursitis"[MeSH] “SLAP”[Text Word] OR “glenohumeral instability”[Text Word] OR "shoulder fractures"[MeSH] OR “proximal humeral”[Text word] OR “humeral head fracture”[Text Word] OR “shoulder dislocation”[MeSH] OR “glenohumeral”[Text Word] OR “arthritis”[MeSH] OR “shoulder arthritis”[Text Word] OR “shoulder osteoarthritis”[Text Word] OR “acromioclavicular”[Text Word] OR “scapula”[Text Word] OR “humerus”[Text Word] OR “clavicula”[Text Word] OR “upper extremity”[Text Word]) AND |
| 2 | ("Surveys and questionnaires"[MeSH] OR “index”[Text Word] OR “scale”[Text Word] OR “score”[Text Word] OR "patient outcome assessment"[MeSH] OR “assessment”[Text Word] OR “evaluation”[Text Word] OR “self report*”[Text Word] OR “patient report”[Text Word] OR “interview-based”[Text Word] OR “personality inventory”[MeSH]) AND ((“Study”[Text Word] OR "psychometrics"[MeSH] OR “psychometr*”[tiab] OR “clinimetr*”[tiab] OR “clinometr*”[tiab] OR "Outcome Assessment, Health Care"[MeSH] OR "outcome assessment"[tiab] OR "outcome measure*"[Text Word] OR "observer variation"[MeSH] OR "Health Status Indicators"[Mesh] OR "reproducibility of results"[MeSH] OR “reproducib*”[tiab] OR "discriminant analysis"[MeSH] OR “reliab*”[tiab] OR “unreliab*”[tiab] OR “valid*”[tiab] OR “coefficient of variation”[Text Word] OR “coefficient”[Text Word] OR “homogeneity”[tiab] OR “homogeneous”[tiab] OR "internal consistency"[Text Word] OR (cronbach*[tiab] AND (alpha[tiab] OR alphas[tiab])) OR “item”[tiab]) OR ((“correlation*”[tiab] OR “selection*”[tiab] OR “reduction*”[tiab])) OR “agreement”[Text Word] OR “precision”[Text Word] OR “imprecision”[Text Word] OR "precise values"[Text Word] OR “test-retest”[tiab] OR (“test”[tiab] AND “retest”[tiab]) OR “stability”[tiab] OR “interrater”[tiab] OR “inter-rater”[tiab] OR “intrarater”[tiab] OR “intra-rater”[tiab] OR “intertester”[tiab] OR “inter-tester”[tiab] OR “intratester”[tiab] OR “intra-tester”[tiab] OR “interobserver”[tiab] OR “inter-observer”[tiab] OR “intraobserver”[tiab] OR “intra-observer”[tiab] OR “intertechnician”[tiab] OR “inter-technician”[tiab] OR “intratechnician”[tiab] OR “intra-technician”[tiab] OR “interexaminer”[tiab] OR “inter-examiner”[tiab] OR “intraexaminer”[tiab] OR “intra-examiner”[tiab] OR “interassay”[tiab] OR “inter-assay”[tiab] OR “intraassay”[tiab] OR “intra-assay”[tiab] OR “interindividual”[tiab] OR “inter-individual”[tiab] OR “intraindividual”[tiab] OR “intra-individual”[tiab] OR “interparticipant”[tiab] OR “inter-participant”[tiab] OR “intraparticipant”[tiab] OR “intra-participant”[tiab] OR “kappa”[tiab] OR “kappa's”[tiab] OR “kappas”[tiab] OR “repeatab*”[Text Word] OR (“measure”[Text Word] OR “measures”[Text Word] OR “findings”[Text Word] OR “result”[Text Word] OR “results”[Text Word] OR “test”[Text Word] OR “tests”[Text Word] OR “generaliza*”[tiab] OR “generalisa*”[tiab] OR “concordance”[tiab] OR (intraclass[tiab] AND correlation*[tiab]) OR “discriminative”[tiab] OR "known group"[tiab] OR "factor analysis"[tiab] OR "factor analyses"[tiab] OR "factor structure"[tiab] OR "factor structures"[tiab] OR “dimension*”[tiab] OR “subscale*”[tiab] OR “(multitrait”[tiab] AND “scaling”[tiab] AND |
| 3 | (analysis[tiab] OR analyses[tiab])) OR "item discriminant"[tiab] OR "interscale correlation*"[tiab] OR “error”[tiab] OR “errors”[tiab] OR "individual variability"[tiab] OR "interval variability"[tiab] OR "rate variability"[tiab] OR (variability[tiab] AND (analysis[tiab] OR values[tiab])) OR (uncertainty[tiab] AND (measurement[tiab] OR measuring[tiab])) OR "standard error of measurement"[tiab] OR “sensitiv*”[tiab] OR “responsive*”[tiab] OR (“limit”[tiab] AND “detection”[tiab]) OR "minimal detectable concentration"[tiab] OR “interpretab*”[tiab] OR ((“minimal”[tiab] OR “minimally”[tiab] OR “clinical”[tiab] OR “clinically”[tiab]) AND (“important”[tiab] OR “significant”[tiab] OR “detectable”[tiab]) AND (“change”[tiab] OR “difference”[tiab])) OR (“small*”[tiab] AND (“real”[tiab] OR “detectable”[tiab]) AND (“change”[tiab] OR “difference”[tiab])) OR "meaningful change"[tiab] OR "ceiling effect"[tiab] OR "floor effect"[tiab] OR "Item response model"[tiab] OR “IRT”[tiab] OR “Rasch”[tiab] OR "Differential item functioning"[tiab] OR DIF[tiab] OR "computer adaptive testing"[tiab] OR "item bank"[tiab] OR "cross-cultural equivalence"[tiab]) AND |
| 4 | AND (“Turkish”[Text Word] OR “Turkish version” OR “Turk*”[Text Word]) |

**Appendix 2**

| Search Strategy in Turkish | Query |
| --- | --- |
| 1 | (“omuz” VEYA “omuz sıkışma sendromu’’ VEYA “omuz ağrısı’’ VEYA “omuz eklemi’’ VEYA “rotator manşet’’ VEYA “omuz hastalıkları’’ VEYA “omuz instabilite’’ VEYA “eklem instabilite’’ VEYA “subakromiyal’’ VEYA “tanımlanmamış omuz ağrısı’’ VEYA “adezif kapsül’’ VEYA “donuk omuz’’ VEYA "bursit" VEYA “SLAP’’ VEYA “glenohumeral instabilite’’ VEYA "omuz kırıkları" VEYA “omuz dislokasyonu’’ VEYA “glenohumeral’’ VEYA “artrit’’ VEYA “omuz artriti’’ VEYA “omuz osteoartriti’’ VEYA “akromiyoklaviküler’’ VEYA “skapula’’ VEYA “humerus’’ VEYA “klavikula“ VEYA “üst ekstremite VE |
| 2 | (“Anketler ve soru ölçekleri’’ VEYA “indeks’’ VEYA “skala’’ VEYA “skor’’ VEYA "hasta sonuç ölçütleri" VEYA “değerlendirme’’ “ölçüm’’ VEYA “kendi raporları’’ VEYA “hasta raporları’’ VEYA “görüşmeye dayalı rapor’’ VEYA “kişilik envanteri’’) VE ((“Çalışma’’ VEYA "psikometrikler" VEYA “psikometre’’ VEYA “klinimetre’’ VEYA “klinometri’’ VEYA "sonuç değerlendirmesi (sağlık hizmeti)" VEYA "sonuç değerlendirmesi" VEYA "sonuç ölçümü" VEYA "gözlemci varyasyonu" VEYA "Sağlık Durumu Göstergeleri" VEYA "sonuçların tekrarlanabilirliği" VEYA “tekrarlanabilir’’ VEYA "ayırt edici analiz" VEYA “güvenilirlik“ VEYA “güvenilmezlik’’ VEYA “geçerli’’ VEYA “varyasyon katsayısı’’ VEYA “katsayı’’ VEYA “homojenlik’’ VEYA “homojen’’ VEYA "iç tutarlılık" VEYA (cronbach VE (alfa VEYA alfalar)) VEYA “madde’’) VE ((“korelasyon’’ VEYA “seçim’’ VEYA “ayırma’’)) VEYA “uzlaşma’’ VEYA “hassasiyet’’ VEYA “belirsizlik’’ VEYA "kesin değerler" VEYA “test-tekrar test’’ VEYA (“test’’ VE “tekrar test’’) VEYA “kararlılık’’ VEYA “yorumlayıcılık’’ VEYA “değerlendirici’’ VEYA “değerlendiriciler arası’’ VEYA “iç değerlendirici’’ VEYA “testçi’’ VEYA “test içi’’ VEYA “test ediciler arası’’ VEYA “gözlemciler arası’’ VEYA “gözlemci içi’’ VEYA “teknisyenler arası’’ VEYA VEYA “inceleyiciler arası’’ VEYA “incelemeler arası’’ VEYA “testler arası’’ VEYA “bireyler arası’’ VEYA “birey içi’’ VEYA “katılımcılar arası’’ VEYA “katılımcı içi’’ VEYA VEYA kappa VEYA kappa's VEYA “tekrarlanabilirlik’’ VE (“ölçüm’’ VEYA “ölçümler’’ VEYA “bulgular’’ VEYA “sonuçlar’’ VEYA “test’’ VEYA “testler’) VEYA “genelleme’’ VEYA “genelleştirme’’ VEYA “uyum’’ VEYA (“sınıf içi’’ VE “korelasyon’’) VEYA “ayırıcı’’ VEYA "bilinen grup" VEYA "faktör analizi" VEYA "faktör analizleri" VEYA "faktör yapısı" VEYA "faktör yapıları" VEYA “boyut’’ VEYA “alt ölçek’’ VEYA (“çok karakterli’’ VE “ölçekleme’’ VE (analiz VEYA analizler) VEYA "madde diskriminant" VEYA "ölçekler arası korelasyon" VEYA “hata’’ VEYA “hatalar’’ VEYA "bireysel değişkenlik" VEYA "aralık değişkenliği" VEYA "hız değişkenliği" VEYA (“değişkenlik’’ VE |
| 3 | (“analiz’’ VEYA “değerleri’) VEYA (“belirsizlik’’ VE (ölçüm) VEYA ölçme)) VEYA "standart ölçüm hatası" VEYA “hassaslık’’ VEYA “duyarlılık’’ VEYA (“limit’’ VE “saptama’’) VEYA "minimal saptanabilir konsantrasyon" VEYA “yorumlanabilirlik’’ VEYA ((“minimal’’ VEYA “minimal olarak’’ VEYA “klinik’’ VEYA “klinik olarak’’) VE (“önemli’’ VEYA “anlamlı’’ VEYA “saptanabilir’’) VE (“değişim’’ VEYA “fark’’)) VEYA (“küçük’’ VE (“gerçek’’ VEYA “saptanabilir’’) VE (“değişim’’ VEYA “fark’’)) VEYA "anlamlı değişiklik" VEYA "tavan etkisi" VEYA "zemin etkisi" VEYA "Öğe tepki modeli" VEYA “IRT’’ VEYA “Rasch’’ VEYA "Farklı öğe işleyişi" VEYA “DIF’’ VEYA "bilgisayar uyarlamalı testi" VEYA "ürün bankası" VEYA "kültürler arası eşdeğerlik") VE |
| 4 | (“Türkçe’’ VEYA “Türkçe Versiyon’’ VEYA “Türk”). |
