# Supplementary material for: Assessment of cross-cultural adaptations and patient-reported outcome measures relevant to shoulder disorders in Turkish: A systematic review using the COSMIN methodology
Source: PLoS One. 2025 May 27;20(5):e0323611. doi: 10.1371/journal.pone.0323611 (PMC12111439; doi:10.1371/journal.pone.0323611)
Supplement: S7 Table — (DOCX) [file pone.0323611.s007.docx]

**S7 Table. Criteria and rating system for cross-cultural adaptation of self-report measures.**

| Steps | Description | Rating Scheme |
| --- | --- | --- |
| Translation | Two (or more) translators should independently translate the original questionnaire. The translators should preferably be native speakers to the target language. | + Translation performed by at least two independent translators  ? Doubtful translation procedure  - Translation performed by only one translator  0 No information about translation |
| Synthesis | The translators should synthesize the multiple translations to produce a consensus of the translations. | + Performed synthesis  ? Doubtful design  0 No information about synthesis OR translation performed by only  one translator |
| Back Translation | Translators, blinded to the original questionnaire, should translate the consensus translation back into the original language. | + Back translation performed by at least two independent translators  ? Doubtful back translation procedure  - Back translation performed by only one translator  0 No information about back translation |
| Expert committee | The expert committee should consolidate all the versions of the questionnaire and develop what would be considered the prefinal version of the questionnaire for testing. | + Clearly reported the existence of an expert committee  ? Doubtful design  0 No information about expert committee |
| Pretesting | The prefinal questionnaire undergoes pilot testing  with members of the target population. | + Performed pretesting  ? Doubtful design  0 No information about pretesting |
