# Supplementary material for: Assessment of cross-cultural adaptations and patient-reported outcome measures relevant to shoulder disorders in Turkish: A systematic review using the COSMIN methodology
Source: PLoS One. 2025 May 27;20(5):e0323611. doi: 10.1371/journal.pone.0323611 (PMC12111439; doi:10.1371/journal.pone.0323611)
Supplement: S8 Table — (DOCX) [file pone.0323611.s008.docx]

**S8 Table. Overview of the GRADE framework and downgrading rules.**

| Quality of Evidence | Lower if |
| --- | --- |
| High | Risk of bias  -1 Serious  -2 Very serious  -3 Extremely serious  Inconsistency  -1 Serious  -2 Very serious  Imprecision  -1 total n=50-100  -2 total n<50  Indirectness  -1 Serious  -2 Very serious |
| Moderate |  |
| Low |  |
| Very low |  |
